# Coevolution of the Human Host and Gut Microbiome: Metagenomics of Microbiota

**DOI:** 10.7759/cureus.26310

**Published:** 2022-06-24

**Authors:** Maryam Shahab, Nimra Shahab

**Affiliations:** 1 Internal Medicine, Queens Internal Medicine and Geriatrics, New York, USA; 2 Internal Medicine, Nowshera Medical College, Nowhsera, PAK

**Keywords:** gut, diversity, dysbiosis, human host, microbiota, microbiome

## Abstract

It is estimated that humans have trillions of microbial cells living in their gut as part of their microbiota. Each human being has an entirely unique microbiome and human gut microbiota composition has been shown to alter with age due to several factors including physical stress, diet, use of antibiotics, prolonged treatments, chronic disease processes, physiological changes, and geographical location. The gut microbiome contributes to overall well-being in a multitude of ways, including digestion, metabolism, immunity, and the creation of vital compounds that the body cannot synthesize on its own. Disequilibrium in the microbiota has been correlated to obesity, heart disease, irritable bowel disease, and certain cancers. The evolution of the human host allowed for the diversity of the microbial community present in the gut. Although previous studies portray the correlation between diet and disequilibrium in host microbiota, the evolutionary dynamics of bacterial commensal flora and the extent to which it is beneficial are still unclear and need additional investigation.

## Introduction and background

It is estimated that each individual has trillions of bacterial cells living in their gut as part of their microbiota [1]. The large intestine of the human gastrointestinal tract (GIT) provides shelter for a vast majority of microorganisms. In particular, the colon contributes to the utmost largest microbial population in the human body, harboring Firmicutes, Bacteroidetes, Actinobacteria, Proteobacteria, Fusobacteria, and Verrucomicrobia [1-3], with Firmicutes and Bacteroidetes accounting for 90% of the total population [2, 3]. Each human being has an entirely unique microbiome [2, 4] and human gut microbiota composition has been shown to alter with age [5-7] due to several factors including stress, diet, use of antibiotics, prolonged treatments, chronic disease processes, physiological changes and geographical location [1-5]. 

The gut microbiome contributes to general health in a multitude of ways, including digestion, immunity, and the creation of vital compounds that the body cannot synthesize on its own [8-10]. Indeed, an unhealthy microbiome has been correlated to a number of chronic ailments, including obesity, asthma, heart disease, diabetes mellitus, irritable bowel disease (IBD), Parkinson's disease, autism, and cancers [1-3, 5, 7-9]. Consequently, adopting a diet that promotes the development of beneficial bacteria in the gut microbiome may lower the risk of acquiring such diseases [3, 5].

It was investigated under what circumstances the gut microbiome evolves and loses its microbial diversity. The longstanding enduring evolutionary consequences experienced by the human host over his or her lifetime were acknowledged and the underlying mechanism responsible for these alterations and microbiota disequilibrium was also identified. 

## Review

The long-term consequence of evolution on the human gut microbiome

Microbiota composition is assumed to begin at birth [1-3, 7, 8]; however, this belief has been called into question after microorganisms were found in the placenta [9]. The GIT is quickly colonized soon after birth as life progresses and faces its challenges including sickness, treatment with antibiotics, and dietary changes producing unstable modifications in the microbiota [8]. The mode of delivery, indeed, influences microbial development [2]. A normal vaginal delivery allows the neonate to encompass a greater composition of *Lactobacilli* [8]. On the contrary, newborns delivered by a caesarian section carry a decreased abundance of *Bacteroides* but are predominated by *Clostridium* species [2].

Compared to breastfed infants, formula-fed newborns are more likely to be infested with *Escherichia coli*, *Bacteroides*, and *Clostridium difficile* [3, 8]. *Bifidobacterium* species have been attributed to lactation and formula milk. Breastfed babies, on the other hand, have a more complex and varied *Bifidobacterium* microbiome than formula-fed newborns [8]. Breastfed neonates have a much more healthy and advantageous intestinal bacterial flora, with a far greater population of *Bifidobacterium* species and less *Clostridium difficile* and *Escherichia coli* than formula-fed infants [2]. 

The microbiomes of the Western hemisphere vary from those of the rest of the world in a number of ways [1, 3, 9-11]. Fifteen to 30% fewer species exist in Western communities than in non-western communities [1]. There is a popular notion that industrialization is causing a "disappearing microbiome" due to the advancing changes in technology and civilization [1, 9]. Certain organisms that are often seen in non-western microbiomes are absent from western microbiomes. Organisms in the *Treponema* and *Brachyspira* species, normally present in many non-western societies, were found to be deficient in the stool samples of the west [9]. In addition, *Firmicutes* and *Proteobacteria* predominate in non-western microbiomes [2], while *Bacteroides* predominate in people living in industrialized settings [9].

There is a great deal of variation in diet between the old world and the new world. In general, diets consisting of high-fiber and low sugar, low fat, and animal protein diet are believed to stimulate the growth of good enteric bacteria in the gut [10, 11]. Because of the widespread adoption of a Western diet, there tends to be a decrease in the overall diversity of human gut flora [1]. There has been an increase in the number of *Firmicutes* and *Enterobacteriaceae* and a reduction in *Actinobacteria* and *Prevotella* genus, respectively, in the enteric microbiome makeup [3]. The GIT is losing its ability to create favorable compounds and anti-inflammatory bacterial species, which is a problem for many individuals [8-10]; these include short-chain fatty acids, butyrate, propionate, acetate, vitamin K, and vitamin B12 [8]. Several bacterial species have proved to be extinct in the bowel of those who eat a Western diet [10]. 

Antibiotic exposure is linked to significant alterations in the makeup of the gut microbiota resulting in long-term consequences; these include medication in the composition, function, and resistance of gut commensal flora [12]. Usage of antibiotics early in life has shown a positive correlation to diabetes mellitus, obesity, coronary artery disease, IBD, asthma, and malignancies due to dysbiosis [12-16]. The positive strong correlation between microbial dysbiosis and the pathogenesis of disease conditions has proved to show dysbiosis as a novel biomarker of disease [11]. Dysbiosis can be defined as ‘a decrease in gut microbial diversity owing to a shift in the balance between commensal and potentially pathogenic microorganisms’ [13] or the ‘loss of beneficial microbial organisms, expansion of pathobionts, and a loss of microbial diversity’ [15].

The overall health and fitness of a human being may be impacted by the interactions between the host and the microbiota. Dysbiosis is accompanied by a cluster of chronic systemic disease conditions that are difficult to treat, namely diabetes mellitus and metastatic carcinoma. Not all these health conditions have a detrimental impact on fitness. Microbiomes are capable of influencing host fitness at numerous phases of life, including reproductive years survival, and fertility [1]. Intestinal bacteria take energy from non-digestible components of milk during infancy, improving nutrition absorption. A stable microbiome throughout childhood provides protection against the entrance of potentially lethal infections by opportunistic microorganisms [17]. Moreover, microbiome coevolution has the capability to disrupt fertility in adults and is thought to play a role in longevity [1]. 

Coevolution of human host and microbes 

Whilst evaluating non-Western to Western microbiomes provides some insight into how industrialized diets and treatments influence the microbial composition, no living species today has an "ancestral" microbiome. Rather, ancient specimens such of fossilized remains, dental calculus, permafrost tissue, and mummified bones provide some insight into how our microbiomes evolved throughout the course of time. Though fossilized bone or mummified intestinal contents provide the finest insights into ancestral gut microbiota composition, such as the impact of modern diet on the host microbiota; yet still, such specimens often do not preserve well, and not all analyzed samples include what we consider gut microorganisms. However, dental calculus retains its composition rather well. The complexity of oral bacterial communities declined with the beginning of agriculture. Interestingly, ancient isolates of the oral microbiome include both documented pathogenic organisms and, perhaps even more intriguingly, potential genes responsible for antibiotic resistance. 

The evolution of the human host allowed for the diversity of the microbial community currently present in the gut. The intestinal bacterial biodiversity of humans is lower than that of our closest living relatives, the African apes; these include chimpanzees and bonobos; however, the number of *Bacteroides* is greater than the abundance of *Methanobrevibacter* and *Fibrobacter* in our closest primates [1]. In comparison to great apes, the composition of intestinal flora seems to have evolved from ancient gut microbes at a faster rate. Cooked food, agricultural development, modern-day industrialization increase in population growth and density, and physical and physiological stresses are some of the characteristics of human evolution and history that may be accountable for the coevolution of the human host and gut microbiota [1, 9]. 

The evolutionary relationship is indicated by the convergence of dietary changes and microbiomes throughout species. Adjustments from a carnivorous diet to an herbivorous diet have quite a particularly big impact on the gut microflora [1, 8]. Species that follow a plant-based diet have gut microbiomes that are comparable to those of their carnivorous and omnivorous relatives yet vary significantly in the genetic composition from other herbivores [1]. Strict carnivores have microbiomes that are taxonomically and functionally comparable to those of herbivores. Nutrition and diet greatly influence the intestinal microbiota within species; both herbivores and carnivores have gut morphological similarities to their common ancestors, which correspond to microbial commonalities [1]. As a result, the correlation between diet, ancestry, evolution, and the microbiome is not always understood and needs further evaluation.

Metagenomics of microbiota

The chief principal microbe found in the intestines is a bacteriophage [2, 3, 13]. The modified genetic makeup of bacteriophage has proved a positive relationship in the pathogenesis of IBD [18], with a marked increase in *Caudovirales* bacteriophage [13]. Phages may bestow differential fitness on genetically susceptible hosts and impact the microbial framework of the GIT by a variety of effects on commensal flora, ‘ranging from cell lysis to the transfer of genetic information encoding toxins or antibiotic resistance’ [13]. Inflammation increases interferon-$\gamma$ (IFN-$\gamma$) production by innate immune cells with consequent production of reactive oxygen species and the loss of microbial diversity [11, 13, 18]. Coevolved microbiota provides a suitable environment for the development of *Candida*, a fungus, further intensifying colonic inflammation via chitin and $\beta$-glucan antigen-presenting cells [13]. Likewise, dysbiotic microbiota is correlated to increased bacteriophage richness and abundance, which may alter the microbiome through the transfer of genetic material. Reduced *Firmicutes* and *Bacteroidetes* and increased *Enterobacteriaceae* have been documented in IBD patients' microbiome [11]. Systemic diseases may develop via coevolution of gut microbiota. One of three underlying mechanisms has been postulated to explain these findings: gain of function dysbiosis, loss of function dysbiosis, and a combination of both gain and loss of function dysbiosis [18]. 

Mononuclear phagocytes, particularly monocytes, macrophages, and dendritic cells, in the pathogenesis of IBD have received a great deal of attention in recent years [11, 13, 18]. Numerous murine models of IBD have indicated that mononuclear phagocytes in the lamina propria play both a protective and morbific role throughout disease development [11, 13]. Three hypotheses have sought to play a role in the underlying mechanism: an incorrect response to beneficial microbes, inadequate clearance of commensal microorganisms leading to prolonged immune activation, and the inability of a pro-inflammatory phenotype to result in inflammatory resolution [11]. Downregulation of tight-junction proteins, which govern paracellular permeability, poor mucus formation owing to goblet cell loss, and altered synthesis of antimicrobial peptides are all consequences of IBD as a result of disrupted intestinal microbiota and epithelium [8, 11, 18]. 

The generation of metabolites by human microbiota have proposed to play a role in the development of IBD [19]. Metabolism of bile acids is one of the primary processes carried out by the human gut microbiome, with a significant impact on the metabolism of host energy [12]. Primary conjugated bile acids are hydrolyzed by bile-salt hydrolase activity of the microbiota [12, 13], allowing them to be more vulnerable to bacterial dysbiosis due to the production of secondary bile acids [8]. An abundance of microbial bile-salt hydrolases in the gut, demonstrate them as a key player in the GIT for digestion [12, 13]. Bacterial bile-salt hydrolases have proven to be a metabolic regulator and have been associated with a decrease in host weight gain, resistance to insulin, and blood cholesterol through the anti-inflammatory activity of bile acid membrane receptors, in particular, nuclear farnesoid-activated X receptor (FXR) and Takeda G protein-coupled receptor 5 (TGR5), a G-protein coupled receptor [12]. The antibacterial properties of secondary bile acid permit them to disrupt the integrity of bacterial cell membranes, and a consequent leak of intracellular contents, hindering the progression of bile acid intolerant microbial organisms [13]. These antagonistic characteristics help determine the composition of intestinal commensal flora while also providing protection to the human species against opportunistic disease conditions [8].

Based on metagenomic analysis, the predominant mediator in the allergic response produced in asthma is believed to be sought from the GIT [11]. Several mouse models have exhibited how the feeding of intestinal microbial flora has been shown to ameliorate allergy symptoms by activated T regulatory cells [11, 13], migrating to the respiratory tract. As a matter of fact, coevolution of dysbiotic microbiota, due to prolonged use of antibiotics, has been associated with the aggravation of asthma in murine. Genetically susceptible neonates and adolescents to asthma have a loss of intestinal microbial bacteria, predominantly *Firmicutes*, including *Faecalibacterium*, *Veillonella*, *Lactobacillus*, as well as *Clostridium* [11]. In addition, the *Firmicutes* dominate the microbiota of obese individuals, with the *Bacteroidetes* population showing a significant decline in number [2]. 

Colorectal carcinoma is the third most common cancer worldwide [18, 20], in addition to being the third most common cause of cancer mortality [2, 21]. *Bacteroidetes* predominate the GIT of those individuals diagnosed with colorectal carcinoma [2]. *Fusobacterium* and *Bacteroidetes* species were proven to be highly related to colorectal cancer using metagenomic testing [13, 18], while *Firmicutes* was deficient [2, 22]. *Fusobacterium* species are believed to promote carcinogenesis and the progression of this neoplasm through an inflammatory-mediated approach [2, 18]. All of these outcomes indicate how changes in colonic microbiota might promote the pathogenesis of colorectal malignancy and metastasis [22]. The implications of these results can potentially lead to techniques for altering intestinal microbes to combat colon cancer and identify persons at increased risk.

Pathogenesis of alteration

The disruption of species-specific intestinal microflora has detrimental effects on health and fitness. Differentiating the processes that underlie different microbial configurations is, therefore, an essential criterion for the therapeutic advantage of the microbiome. Transmission and filtration are two major groups of processes that support microbiome formation [1].

There are two modes of transmission, both being feasible and each with its own evolutionary implications. Vertical transmission is the ‘acquisition of microbes directly from an organism’s parents’, whereas horizontal transmission is the ‘acquisition of microbes from sources other than an organism’s direct parents’ [1]. When host populations deviate, strict vertical transmission triggers symbiont cospeciation with consequential high dependency on interactions between host and gut microorganisms. This impact is a striking feature in obligate intracellular bacteria, which evolved over a long history in insects. This host-microbe relationship has the protentional to progress to coevolution.

Filtration of intestinal microbiota is harbored by two basic underlying mechanisms: microbial competition and habitat filtering, the latter being a critical component of bacterial composition in the gut. The inheritance patterns for the bacteria are thought to be the product of mechanisms such as filtration. Both biochemical and physical variables play a key role in filtering. This constellation of factors includes intestinal pH, intestinal motility, and concentrations of both metabolites and IgA [11, 13]. Metagenomic analyses of microbiota have proven immunological and diet-related genes as significant modulators of gut microbial composition. Genetic techniques aid in determining whether host variables maneuver microbial makeup, either via transmission or filtration. Gut microbiota is crucial for the development of both mucosal and systemic immunity [8]. Metagenomic analyses of microbiota have proven immunological and diet-related genes as significant modulators of gut microbial composition [1]. To some degree, host genetics influence the composition of intestinal microbes and the species richness of universal commensal bacteria.

## Conclusions

It has been documented that the coevolution process of microbiota and the human gut has been occurring for millions of years. The GIT which has trillions of bacteria, all are very beneficial to overall health, fitness, and long-term well-being. These microbes provide a variety of critical functions, including the digestion of carbohydrates in breast milk in newborns and the regulation of the immune system and intestinal health in adults. Consumption of a diverse variety of fiber-rich foods such as fruits, vegetables, whole grains, nuts, seeds, probiotics, and fermented foods helps boost the amounts of these good bacteria in the gut the most effectively. Changes in the fragile habitat of human gut microbiota can alter immune defense, which can lead to the development of chronic illnesses. Furthermore, the positive strong correlation between intestinal dysbiotic bacteria and the underlying mechanism of disease conditions has been documented allowing dysbiosis to be a novel biomarker of disease. Despite previous data exhibiting the relationship between diet and disequilibrium in host-microbiota; the evolutionary dynamics of bacterial commensal flora and to what degree is it beneficial is yet not clear and needs to be further investigated. Moreover, the scope of future research should focus on what external factors influence the human gut virome, rather than the intestinal bacterial community, and what beneficial or adverse effects it has on the human host.
